# A gloomy picture: a meta-analysis of randomized controlled trials reveals disappointing effectiveness of programs aiming at preventing child maltreatment

**DOI:** 10.1186/s12889-015-2387-9

**Published:** 2015-10-18

**Authors:** Saskia Euser, Lenneke RA Alink, Marije Stoltenborgh, Marian J. Bakermans-Kranenburg, Marinus H. van IJzendoorn

**Affiliations:** Centre for Child and Family Studies, Leiden University, P.O. Box 9555, 2300 RB Leiden, Netherlands

**Keywords:** Intervention, Prevention, Meta-analysis, RCT, Child maltreatment

## Abstract

**Background:**

Consistent findings about the effectiveness of parent programs to prevent or reduce child maltreatment are lacking.

**Methods:**

In the present meta-analysis we synthesized findings from 27 independent samples from randomized controlled trials (RCTs) on the effectiveness of 20 different intervention programs aimed at (i) preventing the occurrence of child maltreatment in the general population or with at-risk but non-maltreating families, or (ii) reducing the incidence of child maltreatment in maltreating families.

**Results:**

A significant combined effect on maltreatment (*d* = 0.13; *N* = 4883) disappeared after the trim-and-fill approach that takes into account publication bias against smaller studies without significant outcomes. However, moderator analyses showed that larger effect sizes were found for more recent studies, studies with smaller samples, programs that provide parent training instead of only support, programs that target maltreating instead of at-risk families, and programs with a moderate length (6–12 months) or a moderate number of sessions (16–30).

**Conclusions:**

More RCTs are needed to further unravel which factors are associated with program effectiveness. Because currently existing programs appeared to only reduce and not prevent child maltreatment, efforts in the field of preventive intervention should also focus on the development and testing of preventive programs for families at risk for child maltreatment.

**Electronic supplementary material:**

The online version of this article (doi:10.1186/s12889-015-2387-9) contains supplementary material, which is available to authorized users.

## Background

The number of parent support programs aimed at preventing or reducing child maltreatment has grown over the last decades. Some of these programs were found to have a positive impact on various parenting domains in studies using randomized controlled designs (RCTs; [1]). However, consistent findings about the effectiveness of such programs to prevent or reduce child maltreatment are lacking [2, 3]. The current meta-analysis aims to fill this gap. We synthesized findings of all randomized controlled trials (23 studies) that tested the effectiveness of 20 different programs, aimed at the general population, at-risk, and maltreating groups, in order to reveal the overall success of programs to prevent or reduce the occurrence of child maltreatment and to uncover factors that influence the effectiveness of intervention programs.

### Child maltreatment

A recent series of meta-analyses indicated that child maltreatment is a serious problem, affecting children all over the world. Worldwide prevalence rates of different types of maltreatment ranged from 0.3 % based on studies with reports from professionals to 36.3 % based on self-report studies [4]. Risk factors for child maltreatment are low socio-economic status, parental mental health problems, family isolation, and single parenthood [5–7]. Child maltreatment is associated with short-term and long-term negative consequences. Victims have an increased risk for physical, behavioral, and psychological problems, also up into adulthood (e.g., [8–11]), and benefit less from treatment compared to non-maltreated individuals [12], leading to high costs for individuals and society. Given the high prevalence rates and serious consequences of maltreatment, effective prevention and reduction of child maltreatment is essential.

### Intervention programs

Over the last decades, the number of parent support programs has increased exponentially [1]. Most of these programs are targeted and provide support to a clearly defined population identified on the basis of risk factors for child maltreatment. However, some programs are available for everyone or at least for a large proportion of the population. Examples of such universal programs are Triple-P [13] and SOS! Help for Parents [14]. These programs aim to prevent the occurrence of child maltreatment in the general population, for example by using the media to inform parents about effective parenting strategies or by providing a short parent skill training to parents who visit a well-baby clinic. Concerning programs that target a clearly defined population, programs that *prevent* the occurrence of child maltreatment in at-risk, but non-maltreating families, can be distinguished from programs that *reduce* the incidence of child maltreatment in maltreating families.

A well-known targeted *prevention* program is the Nurse-Family Partnership developed by Olds and colleagues (e.g., [15, 16]). This program specifically targets pregnant adolescent women who are unmarried and/or have a low income, but women without any of these risk factors are also allowed to participate in the program. It consists of nurse home visits in the prenatal period and during the first two years of the child's life. The nurses promote improvement of the women's health behavior during and after pregnancy, help building supportive relationships with family and friends, and link them with other needed services. The Elmira (New York) trial indicated a significant difference of 80 % fewer child maltreatment cases in the intervention group compared to the control group during the period of intervention. However, these positive results disappeared in the two years after the end of the program [17].

Parent–child Interaction Therapy (PCIT) is an example of a targeted program that aims to *reduce* the incidence of child maltreatment in physically abusive parents. Families receive 14 weekly one-hour live-coached sessions of parent–child interaction training. The training consists of child-directed interaction, in which the parent is instructed to follow the child's lead, and parent-directed interaction in which the parent is taught to direct the child's behavior and use consistent disciplinary techniques [18]. Several studies have shown that PCIT indeed effectively reduces child behavior problems [18, 19], and an RCT also indicated significantly fewer reports of physical abuse and improved parenting skills in the PCIT condition compared to families who received community services [20].

### Prior meta-analytic findings

A number of meta-analyses have synthesized results on the effectiveness of intervention programs aimed at preventing or reducing child maltreatment. However, some meta-analyses did not specifically include papers that measured the actual occurrence of child maltreatment [21, 22], focused solely on non-maltreating families [23–25], included only home-visiting programs [23, 25, 26], and/or included studies with less rigorous designs than RCTs [21, 23, 24]. For instance, Layzer and colleagues [21] combined abuse and neglect outcomes with child injuries, accidents, and removal from the home into a single category 'child safety', which makes it impossible to estimate the actual ability of programs to prevent or reduce child maltreatment. Geeraert and colleagues [24] examined the effect of early prevention programs on actual abuse and neglect, but they included mostly nonrandomized designs. A significant but small overall effect on reported child maltreatment was found, but moderator analyses were not conducted. Similarly, Filene and colleagues [23] examined the effect of home visiting programs on child maltreatment, but they also included nonrandomized designs, and did not include maltreating families, thereby only examining the preventive effect of interventions. In contrast to Geeraert and colleagues, these authors did not find a significant effect on child maltreatment. In another meta-analysis, only RCTs were included, but the focus of this meta-analysis was solely on programs starting during pregnancy or within 6 months after birth [22]. It revealed a small but significant effect for maltreatment outcomes at the end of intervention, but no effect at follow-up. The only significant moderator that was identified for child abuse and neglect measures was year of publication; more recent studies yielded smaller effect sizes.

### The current study: Program effectiveness and moderators

The current meta-analysis aims to estimate the average effect of intervention programs that provide services to parents in order to prevent or reduce child maltreatment. We only included RCTs, in which participants are fully randomly assigned to either the intervention or the control condition. Because of the random assignment, it can be assumed that the two groups do not differ systematically before the start of the program. Clustered randomized trials were excluded, because participants are not fully randomly assigned and therefore participants (or their contexts) in one cluster may not be comparable to participants in other clusters. Further, we aimed to include three types of programs: those targeting the general population, aimed at *preventing* maltreatment, those for families at risk for child maltreatment, aimed at *preventing* maltreatment, and those specifically developed for maltreating families, aimed at *reducing* maltreatment. We only included studies if they reported on actual maltreatment outcomes and used this outcome in our meta-analysis. Child maltreatment was defined as “any act or series of acts of commission or omission by a parent or other caregiver that results in harm, potential for harm, or threat of harm to a child” (Centers for Disease Control and Prevention (CDC)). In addition, we examined whether various intervention, design, sample, and study characteristics were associated with program effects.

#### Intervention characteristics

An important characteristic of the intervention is the focus of the program. In some programs, parents receive various sorts of support (e.g., social, emotional, material) in order to build on strengths and improve overall family functioning, without actual parenting skills training. For example, in Healthy Families America parents receive support to reduce social isolation, access recourses such as food, housing, employment, and health care, and improve their knowledge about child development [27]. Other programs do provide actual training for parents to improve their parenting skills, such as SOS! Help for parents [14], in which parents are instructed about (the role of) parenting skills and common mistakes in parenting, or Parent Child Interaction Therapy [20], in which parents receive (among other things) live parent–child coaching sessions to improve parent–child interaction skills. Finally, some intervention programs combine parent training and support. For example, in the Project Support intervention [28], mothers are taught skills for child behavioral management by instruction, practice, and feedback, and they are provided with instrumental and emotional support, such as training in how to evaluate a child care provider.

Further, the way of delivery is another intervention characteristic that can differ substantially between programs. Some programs use support-groups in a center-based setting [29], others consist of personal home visits [15] or combine center-based and home-based sessions [30]. The number of sessions and the duration varies from program to program. For instance, in the Nurse-Family Partnership Program [15], parents receive 45 home visits during the first two years of the their child's life, while the SOS! Help for parents program (SOS) described by Oveisi and colleagues [14] consists of only two 2-h weekly sessions. A meta-analysis on the effectiveness of interventions aimed at improving parental sensitivity and parent–child attachment revealed that programs with fewer contacts were more effective in improving sensitivity and attachment [31], but it is unclear if this is also true for programs aimed at preventing or reducing child maltreatment. Last, and more specific for programs aimed at preventing child maltreatment, the moment of onset of the program, and thus the age of the child at the start of the program, has been discussed as an important moderator of a program's effectiveness. Although it has been suggested that programs for the prevention of child maltreatment would be most effective if starting before birth [1], meta-analytic evidence showed that programs focusing on parental sensitivity or parent–child attachment that started 6 months after birth were at least as effective as programs with an earlier onset [31].

#### Sample characteristics

Intervention programs target different populations. Universal programs target the general population, while targeted programs focus on a clearly defined group of families at risk for child maltreatment or maltreating families. Some have suggested that programs with a clear target population would be more effective [32]. This may be especially true for programs that target maltreating families, because those families show the behaviors that are targeted for change, and therefore they may have the greatest potential for demonstrating change.

#### Design characteristics

The rigor of the study design may also affect the effect size. Studies with poorer methodological designs likely yield larger effect sizes [3]. The use of intent-to-treat analyses is an example of a methodological strength, as selective refusals after randomization or selective attrition during the intervention may affect the randomization. In intent-to-treat analyses, group differences are analyzed based on the original random assignment. Other design characteristics are sample size, whether assessment was blind for group assignment and whether a pretest was included. Moreover, the type and amount of services received in the control condition differs between programs. Largest effect sizes may be expected when the control group received few or even no services. In addition, there may be differences in effect sizes for short-term and long-term effects. On the one hand, it may be expected that intervention effects decrease or even disappear over time. On the other hand, there may be sleeper effects, meaning that intervention effects increase over time, because parents would need some more time to practice new skills [33]. Finally, the method of assessment of child maltreatment may influence effect sizes. Although self-report measures may be informative since participants may know their own experiences best, self-reports have several disadvantages. Participants may interpret definitions of maltreatment or parenting practices differently than researchers and it may be difficult for participants to remember the exact frequency of specific events in the past. In addition, self-report of maltreatment experience is not possible in early childhood. In contrast, reports from professionals who work with children do cover all ages and these reports are generally coded by expert coders who use the same set of definitions. The downside of this method is that professionals may not be aware of all cases of maltreatment; they may only see the tip-of-the-iceberg [34].

## Methods

### Literature search

Eligible studies were identified using a systematic search in three electronic databases (Web of Science, ERIC, and PsychInfo) using the terms *child abuse* and/or *child neglect* and/or *child maltreatment* combined with interven* or preven*, and written in English. Studies published up until the end of 2012 were identified, and no earliest time point was specified. Articles were included if they described (1) empirical studies in which (2) a randomized controlled trial (RCT) was applied to test the effectiveness of (3) an intervention program for the general population, families at risk for maltreatment or maltreating families with child maltreatment as outcome measure (4) in non-clinical samples. The Child Abuse Potential Inventory (CAPI) was not included as a measure of maltreatment (e.g., 35]). Articles were excluded if not enough information was provided for calculation of effect sizes. For instance, the number of families that received the Triple P intervention in the population based trial described by Prinz and colleagues [36] is unclear, making it impossible to calculate an effect size. Another reason for excluding this Triple P trial was the use of clustered randomization. If publications reported on the same sample or on overlapping samples, the publication providing the maximum of information was included in the meta-analysis. Thus, the independence of samples and the inclusion of every participant only once in the meta-analysis were ascertained. When possible and necessary, the coding form for the study was supplemented with information from the excluded publication on the same sample.

**Fig. 1:**
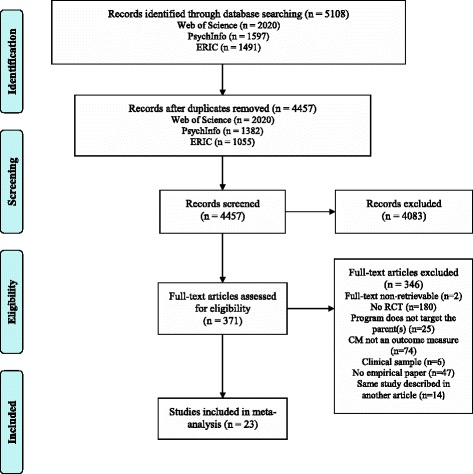
Overview of articles included and excluded in the meta-analysis

The initial search procedure identified 5108 articles. First, 651 duplicate articles were excluded. After screening the abstracts of the remaining articles, another 4083 articles were excluded, because they did not meet inclusion criteria. From the 374 full-text articles assessed for eligibility, 349 articles were excluded from the meta-analysis because they did not meet the inclusion criteria or were non-retrievable (see Fig. 1 for a flow chart and Additional file 1 for the PRISMA checklist). The final sample of 23 eligible articles covered reports on 27 independent samples with maltreatment outcomes in the general population, at-risk and maltreating groups (*N* = 4883 families; see Table 1).

**Table 1:** Intervention studies included in the meta-analysis

Author(s)	Intervention	Age child (years)^a^	Focus	Duration (months)	Sessions	Setting	Delivery	Type of sample	*N*
Barth (1991 [63])	CPEP	−0.3	S	6	11	H	I	At risk	191
Brayden (1993 [29])	Maternal and child health	−0.4	S	29	14	C	G & I	At risk	263
Bugental (2002 [56])	Healthy Start	−0.2	S	12	20	H	I	At risk	48
Bugental (2002 [56])	Healthy Start +	−0.2	TS	12	20	H	I	At risk	49
Bugental (2009 [64])	Healthy Start +	0.2	TS	12	17	H	I	At risk	110
Bybee (1986 [65])	Family Diversion	NR	TS	4	57	H	I	Maltreating	31/27^2^
Chaffin (2004 [20])	PCIT	8.0	T	6	23	C	G & I	Maltreating	60
Chaffin (2004 [20])	EPCIT	8.0	TS	6	30	C	G & I	Maltreating	51
Chambliss (1998 [66])	Healthy Families	0.0	S	12	52	H & C	G & I	At risk	249
Dakof (2010 [54])	EMP	NR	O	14	26	NR	I	At risk	62
DePanfilis (2005 [43])	Family Connections	8.3	O	6	24	H	I	At risk	154
Duggan (2004 [67])	Healthy Start	0.0	S	36	NR	H	I	At risk	561
Duggan (2007 [57])	Healthy Families	0.0	S	24	42	H	I	At risk	268
DuMont (2008 [46])	Healthy Families	0.0	S	24	36	H	I	At risk	992
Fergusson (2005 [68])	Early Start	0.0	S	36	NR	H	I	At risk	391
Jouriles (2010 [28])	Project Support	5.4	TS	8	22	H	I	Maltreating	35
Lam (2009 [44])	BCT	9.0	O	3	24	C	I	At risk	15
Lam (2009 [44])	PSBCT	8.9	T	3	24	C	I	At risk	15
LeCroy (2011 [69])	Healthy Families	0.0	S	12	NR	H	I	At risk	171
MacMillan (2005 [70])	Nurse Home visiting	5.1	S	24	46	H	I	Maltreating	160
McIntosh (2009 [71])	Family Partnership	−0.5	T	18	78	H	I	At risk	122
Olds (1986 [15])	Nurse Home visiting	−0.3	S	3	9	H	I	At risk	167
Olds (1986 [15])	Nurse Home visiting	−0.3	S	27	45	H	I	At risk	176
Oveisi (2010 [14])	SOS	4.4	T	1	2	C	I	Population	224
Silovsky (2011 [72])	SafeCare +	0.0	S	NR	NR	H	I	At risk	105
Stevens-Simon (2001 [73])	CAMP with Home visiting	−0.4	O	24	30	H & C	G & I	At risk	127
Swenson (2010 [30])	MST-CAN	13.8	TS	8	NR	H & C	I	Maltreating	86

*Note*. *CPEP* Child Parent Enrichment Project, *Healthy Start +* Enhanced Health Start Program, *PCIT* Parent Child Interaction Therapy, *EPCIT* Enhanced Parent Child Interaction Therapy, *EMP* Engaged Moms Program, *BCT* Behavioral Couples Therapy, *PSBCT* Parent Skills Behavioral Couples Therapy, *CAMP* Colorado Adolescent Maternity Program, *MST-CAN* Multisystemic Therapy for Child Abuse and Neglect, *NR* Not reported, *S* Support, *T* Parent training, *TS* Parent training and support, *O* Other, *H* Home, *C* Center, *I* Individual, *G* Group

^a^A negative age indicates that the intervention started during pregnancy

Because of the strict inclusion criteria, some effectiveness studies testing well-known interventions programs were not included in this meta-analysis. For instance, for the Nurse-Family Partnership program, we only included the Elmira trial, and excluded the Denver and Memphis trial, because in these trials child maltreatment was not included as an outcome measure [37]. The same was true for a second PCIT trial [38] in which only risk factors for maltreatment were reported, and for an AF-CBT trial [39] with professional satisfaction and knowledge as outcome. Another reason for exclusion was the use of non-fully random assignment to intervention and control conditions, such as including new participants to the intervention or control condition after randomization was completed. In addition, three studies evaluating the SafeCare [40] or SEEK program [41, 42] were excluded because clustered randomization was used.

### Coding system

A standardized coding system was used to rate each study on sample, intervention, and design characteristics (Table 2). *Background moderators* were year of publication and type of publication (journal article or dissertation). *Sample characteristics* were continent of origin, type of sample (general population, high-risk for maltreatment, maltreating), and ethnicity (majority or minority).

**Table 2:** Coding system

Variable	Coding	Description
*Intervention characteristics*		
Name of the program		
Focus	1. Support	
	2. Parent training	
	3. Parent training and support	
	4. Other	
Location of delivery	1. At home	
	2. At a center	
	3. Both	
Delivery format	1. Individual	
	2. In a group	
	3. Both	
Duration	1. < 6 months	
	2. 6–12 months	
	3. > 12 months	
Sessions	1. < 16	
	2. 16-30	
	3. > 30	
Age child at start intervention		Continuous; if a range was provided, the minimum age was coded
*Sample characteristics*		
Continent of origin	1. Australia	Including New Zealand
	2. North America	Including USA and Canada
	3. Europe	
	4. Africa	
	5. South America	
	6. Asia	
Type of sample	1. General population	
	2. At risk for maltreatment	
	3. Maltreating	
Ethnicity	1. Majority	Percentage of each category in the sample, based on reports in the study
	2. Minority	
*Design characteristics*		
Sample size		Continuous
Response rate		Continuous
Intent to treat	1. Yes	
	2. No	
Blind assessment	1. Yes	
	2. No	
Pre-test	1. Yes	
	2. No	
% Active at the end of the intervention		Continuous
Control condition	1. No active intervention elements	
	2. Service as usual	
	3. Other…	
Timing follow-up		Continuous
Type of measure	1. Self-report	
	2. Other-report	
*Background characteristics*		
Year of publication		Continuous
Type of publication	1. Journal article	
	2. Dissertation	

*Intervention characteristics* consisted of name of the program, focus of the intervention, control condition, location of delivery, delivery format, duration, number of sessions, and age child at onset of intervention. If the content of the intervention and control group were identical [43], the difference in duration and number of sessions between the intervention and control condition were coded.

*Design characteristics* included sample size, response rate, use of intent-to-treat analyses (yes/no), blind assessment (yes/no), inclusion of a pre-test measure (yes/no), percentage of participants actively involved in the program by the end of the intervention, and services for the control group. Further, we coded the timing of the post-intervention assessment as the time interval between the end of intervention and the follow-up (in months). We included separate effect sizes for one study if more than one follow-up was reported. For instance, Lam and colleagues [44] reported child maltreatment outcomes directly after the intervention (time point 0), half a year later (time point 6), and a year after termination of the intervention (time point 12). However, to ensure the independence of samples, only the first follow-up was included to test the overall effect. If results during the intervention as well as results at the end of intervention were reported, only post-test results were included in the meta-analysis. If only results during the intervention were reported, timing of post-intervention assessment was coded as zero and duration of the intervention was adjusted to the timing of assessment. Finally, we coded type of measurement (self-report or other report). If results for more than one type of maltreatment were reported, effect sizes were meta-analytically combined into one effect size, Cohen's *d*.

All studies were coded independently by two coders. Coders achieved good reliability, intraclass correlations ranged from .65 to 1.00 (*M* = .96, *k* = 14 studies), and kappas ranged from .55 to 1.00 (*M* = .81, 91 % agreement, *k* = 14 studies). Disagreements were discussed and final scores reflected the consensus of the two coders. To group the interventions based on their focus, four experts independently sorted the 23 intervention programs into four different sets. To obtain a final sorting of the intervention programs, programs clustered by the majority of the experts were considered a category. This final sorting led to four different categories: Support, Parent training, Parent training combined with support, and Other.

### Data analyses

The meta-analysis was performed using the Comprehensive Meta-Analysis (CMA) program [45]. For each study, the outcome was transformed into Cohen’s *d*. Study effect sizes indicate post intervention differences on child maltreatment between the intervention and control group. No outliers were found for study effect sizes. Combined effect sizes were computed using CMA. One outlying sample size [46] was winsorized, by replacing it with a marginally lower score, while remaining the largest sample size in the set of studies.

Significance tests and moderator analyses were performed through random effects models. In contrast to fixed effect models, random effects models allow for the possibility that there are random differences between studies that are not associated with sampling error and thus point to different study populations [47, 48]. Q-statistics were computed to test the heterogeneity across studies. In addition, we computed 95 % confidence intervals (CIs) around the point estimate of each set of effect sizes. Q-statistics and p-values were also computed to assess differences between combined effect sizes for specific subsets of study effect sizes grouped by moderators. Contrasts were only tested when at least two of the subsets consisted of at least four studies [31]. For continuous moderators, Fisher’s Z-scores were used in weighted least squares meta-regression analyses.

We used the “trim and fill” method [49, 50] to calculate the effect of potential data censoring or publication bias on the outcome of the meta-analysis. Using this method, a funnel plot is constructed of each study’s effect size against the sample size or the standard error (usually plotted as 1/SE, or precision). It is expected that this plot has the shape of a funnel, because studies with smaller sample sizes and larger standard errors have increasingly large variation in estimates of their effect size as random variation becomes increasingly influential, whereas studies with larger sample sizes have smaller variation in effect sizes [50, 51]. The plots are expected to be shaped like a funnel if no data censoring is present. However, since smaller non-significant studies are less likely to be published (the ‘file-drawer’ problem, [52]), studies in the bottom left hand corner of the plot are often omitted [51]. With the “trim and fill” procedure the k right-most studies considered to be symmetrically unmatched are trimmed and their missing counterparts are imputed or “filled” as mirror images of the trimmed outcomes. This then allows for the computation of an adjusted overall effect size and confidence interval [53].

## Results

### Which interventions are most effective?

**Fig. 2:**
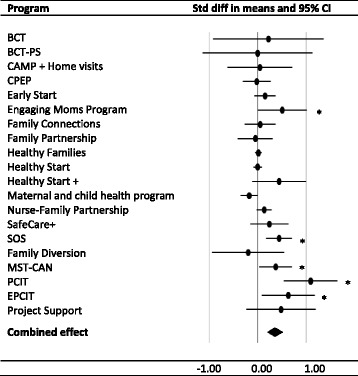
Forest plot for interventions with effects on child maltreatment in the general population, at risk, and maltreating groups. *p < .05. *Note*. BCT = Behavioral Couples Therapy; BCT-PC = Parent Skills Behavioral Couples Therapy; CAMP = Colorado Adolescent Maternity Program; CPEP = Child Parent Enrichment Project; Healthy Start + = Enhanced Health Start Program; MST-CAN = Multisytemic Therapy for Child Abuse and Neglect; PCIT = Parent Child Interaction Therapy; EPCIT = Enhanced Parent Child Interaction Therapy

Twenty different intervention programs were tested in the studies included in the meta-analysis. The effect sizes are shown in a forest plot in Fig. 2. Of the 20 different intervention programs focusing on maltreatment outcomes only five (25 %) programs effectively prevented or reduced child maltreatment (Engaged Moms Program [EMP], [54]; SOS, [14]; Multisystemic Therapy for Child Abuse and Neglect [MST-CAN], [30]; and PCIT with and without individualized services, [20]). PCIT in its original form [20] yielded the largest effect size (*d* = 1.09).

### Combined intervention effect

**Fig. 3:**
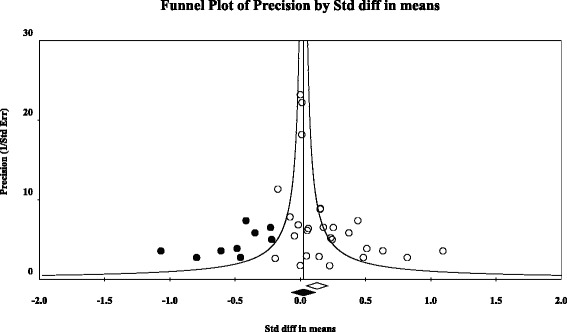
Funnel plot of intervention studies with effects on child maltreatment in the general population, at risk, and maltreating groups. *Note.* White circles indicate observed studies and black circles indicate imputed studies

The combined effect size of the 27 intervention effects on maltreatment in the general population, families at-risk for maltreatment or maltreating families was *d* = 0.13 (*N* = 4883; 95 % *CI*: 0.05 ~ 0.21; *p* < .01), in a heterogeneous set of outcomes (*Q* = 56.06, *p* < .01). The trim-and-fill approach showed that 9 studies should be trimmed and filled (Fig. 3), with a resulting non-significant adjusted combined effect size of *d* = 0.02 (95 % *CI*: −0.06, 0.11). This pattern of results suggests publication bias favoring the publication of smaller studies with significant findings.

### Moderator analyses

Although no significant combined effect was found, moderator analyses indicated significant differences in effects among subsets of studies. Results of the moderator analyses are shown in Table 3. The moderator analysis for focus of the intervention program showed a significant contrast: programs with a focus on parent training, either with (*d* = 0.37) or without support (*d* = 0.37) were significantly more effective than programs that solely provide support (*d* = 0.03), *Q*(3) = 15.85, *p* < .01. Furthermore, interventions with a moderate number of sessions (16–30; *d* = 0.37) were significantly more effective compared to interventions with fewer (*d* = 0.05) or more sessions (*d* = 0.03), *Q*(2) = 9.65, *p* < .01. The moderator analysis for duration of the intervention showed comparable results: only interventions with a duration of 6–12 months yielded significant effect sizes (*d* = 0.23), whereas interventions with a duration shorter than six months (*d* = 0.22) or longer than twelve months (*d* = 0.04) did not significantly reduce child maltreatment, *Q*(2) = 6.04, *p* < .05. The large majority of the studies (*n* = 23; 85 %) were conducted in the USA. Because the four studies from outside the USA were all from different countries, the moderating effect of the country of origin of the sample could not be tested. However, no significant contrast was found between samples originating from the USA and samples from other countries. Type of sample was a significant moderator of the effect size. Interventions were significantly more effective in maltreating samples (*d* = 0.35) than in at-risk samples (*d* = 0.05), *Q*(1) = 9.31, *p* < .01. Moderator analyses for any of the other intervention, sample, or design characteristics did not show significant contrasts.

**Table 3:** Combined effect sizes and moderator analyses^a^ for intervention effects

	*K* ^b^	*N*	*d*	*95 % CI*	*Q homogeneity*	*Q* ^c,d^ *contrast*
Total effects on maltreatment	27	4883	0.13**	0.05 ~ 0.21	56.06**	
*Intervention characteristics*						
Focus						
Support	13	3742	0.03	−0.04 ~ 0.11	13.28	15.85**
Parent training	4	421	0.37**	0.15 ~ 0.59	12.45**	
Parent training and support	6	362	0.37**	0.16 ~ 0.59	5.21	
Other	4	358	0.17	−0.09 ~ 0.44	2.39	
Location of delivery						1.97
At participants’ home	17	3731	0.10	−0.00 ~ 0.19	15.19	
In center	6	628	0.26*	0.05 ~ 0.46	32.16***	
Both	3	462	0.10	−0.15 ~ 0.36	4.47	
Delivery format						0.16
Individual	22	4133	0.14**	0.05 ~ 0.23	30.19	
Individual and group	5	750	0.09	−0.11 ~ 0.29	24.51***	
Duration						6.04*
< 6 months	5	452	0.22	−0.01 ~ 0.45	4.99	
6-12 months	11	1204	0.23**	0.10 ~ 0.37	25.01**	
> 12 months	10	3122	0.04	−0.06 ~ 0.13	12.55	
Sessions						9.65**
< 16	4	845	0.05	−0.12 ~ 0.23	14.57**	
16-30	11	726	0.37***	0.19 ~ 0.56	15.47	
> 30	7	1998	0.03	−0.10 ~ 0.16	3.51	
*Sample characteristics*						
Country of origin						0.66
USA	23		0.11*	0.02 ~ 0.20	45.46**	
Other	4		0.19*	0.01 ~ 0.37	5.30	
Type of sample						9.31**
At risk	20	4236	0.05	−0.02 ~ 0.12	21.85	
Maltreating	6	423	0.35***	0.17 ~ 0.53	13.18*	
General population	1	224	0.44**	0.13 ~ 0.75		
*Design characteristics*						
Intent to treat						1.86
Yes	20	3349	0.17**	0.07 ~ 0.27	37.19**	
No	7	1534	0.05	−0.09 ~ 0.19	16.23*	
Blind assessment						2.33
No	18	3369	0.19**	0.08 ~ 0.29	42.50**	
Yes	9	1514	0.06	−0.07 ~ 0.18	12.33	
Pre-test						1.55
No	18	3945	0.09*	0.01 ~ 0.18	38.87**	
Yes	9	938	0.20**	0.06 ~ 0.35	9.99	
Control condition						1.85
Other	16	2661	0.18**	0.05 ~ 0.30	30.74*	
Treatment as usual	7	1011	0.05	−0.12 ~ 0.21	11.36	
Type of measure						3.08
Self-report	9	1085	0.31**	0.12 ~ 0.49	6.13	
Other report	13	1764	0.11	−0.02 ~ 0.24	30.36**	
*Background characteristics*						
Type of publication						
Journal article	25	4603	0.14**	0.06 ~ 0.23	54.53***	
Dissertation	2	280	−0.10	−0.42 ~ 0.23	0.08	

*k* number of study outcomes, *N* total sample size, *d* effect size (Cohen’s *d*), 95 % *CI* 95 % confidence interval around the point estimate of the effect size, *Q*_homogeneity_ homogeneity statistic, *Q*_contrast_ moderation statistic

*p < .05, **p < .01, ***p < .001

^a^Only categorical moderators are included in the table; the effects of continuous moderators are described in the text

^b^Missings were excluded from moderator analyses. Therefore, sample sizes range from 22 to 27

^c^Subgroups with k < 4 excluded from contrast

^d^After controlling for year of publication and sample size, only type of intervention remained a significant moderator

Meta-regression analyses with one predictor at a time revealed that intervention effects were significantly moderated by year of publication (*z* = 2.11, *p* < .05, *k* = 27) and sample size (*z* = −2.83, *p* < .01, *k* = 27). Studies that were published more recently and studies with smaller sample sizes yielded larger effect sizes. Furthermore, age of the child at the start of the intervention yielded a significant positive regression weight (*z* = 4.27, *p* < .001, *k* = 27), indicating that interventions targeting families with older children had larger effects. Neither socioeconomic status nor ethnicity of the sample significantly influenced the effectiveness of interventions.

### Multivariate analyses

We conducted multiple meta-regression analyses to test whether the moderating effects of focus, type of sample, age of the child, number of sessions or duration of the intervention were confounded by the background characteristics year of publication and/or sample size. In the first model, we included year of publication and sample size. Both year of publication (*z* = 2.53, *p* < 0.05, *k* = 27) and sample size (*z* = −2.83, *p* < .01, *k* = 27) were significant predictors of the effect size. Next, the five significant moderators were added separately to the model. Focus was added as a dichotomous variable, indicating the difference between support only and training with or without support. When controlled for the two background variables, program focus was a significant predictor of intervention effectiveness (*z* = 3.00, *p* < .01, *k* = 23). Interventions that provide parent training yielded larger effect sizes compared to interventions that provide only support. Type of sample was not significant (*z* = 1.90, *p* = .06, *k* = 26), indicating that controlling for year of publication and sample size, interventions targeting maltreating samples did not yield larger effect sizes compared to at risk samples.

The effect of child age at the start of the intervention failed to be a significant moderator when year of publication and sample size were taken into account (*z* = 1.74, *p* = .08, *k* = 27). In the next regression, number of sessions and duration of the intervention were both added as two dummy variables. The first dummy indicated the difference between interventions in the first category (< 16 sessions or < 6 months) versus the other two categories, and the second dummy indicated the difference between the middle category (16–30 sessions or 6–12 months) versus the first and last category. On top of the two background characteristics, neither the duration (*p*s > .14) nor the number of sessions of the intervention (*p*s > .14) significantly predicted the effect size.

### Long term effects

Six studies included more than one follow-up. To examine the possible long term effects of interventions, the difference between the effect sizes at the first and last follow-up was calculated, such that a positive difference indicated a larger effect at the last assessment compared to the first assessment. The combined effect size for the six difference scores was not significant (*d* = 0.13 [*N* = 530; 95 % *CI*: −0.04 ~ 0.31; *p* = 0.14], in a homogeneous set of outcomes [Q = 2.34, p = .80]), indicating that the effect of interventions on reducing or preventing child maltreatment did not change over time. However, this finding is based on only six studies and should therefore be considered as exploratory.

## Discussion

Contrary to our expectations, the current meta-analysis did not show significant combined effects of intervention programs in randomized controlled trials on the reduction or prevention of child maltreatment in the general population, at-risk or maltreating families. Taking into account the presence of publication bias against smaller studies with non-significant results in this research domain, we failed to find a significant overall effect.

We did find, however, a subset of studies with promising intervention effects. First of all, differences between intervention studies in effect sizes could be partly explained by the focus of the intervention program. Programs that solely provide support, such as promoting healthy behaviors during pregnancy and early parenthood [29], establishing social support networks [56], or screening for developmental delay [57] were not effective. In contrast, we found significant effects for intervention programs offering parent training, regardless whether additional support is provided or not. This subgroup of intervention programs includes, among others, Multisystemic Therapy for Child Abuse and Neglect [30] and Parent Child Interaction Therapy (with and without additional individualized services; [20]), which were both effective in reducing child maltreatment. Secondly, we found significant intervention effects in maltreating samples, but not in at-risk samples, indicating that programs are only effective in reducing (but not preventing) child maltreatment. Although previous reviews have suggested limited effectiveness of programs in reducing child maltreatment (e.g., [58, 59]), the current study is, to our knowledge, the first to meta-analytically compare programs to prevent and reduce child maltreatment.

The *'less is more'* effect in attachment-based interventions found by Bakermans-Kranenburg and colleagues [31] seems only partly applicable to programs aimed at reducing or preventing child maltreatment. We found a curvilinear association with program duration and number of program sessions. Programs with a moderate duration (6–12 months) or a moderate number of sessions (16–30) yielded significantly higher effect sizes compared to shorter or longer programs and programs with fewer or more sessions. This indicates that for at-risk or maltreating parents who are targeted in these interventions somewhat more comprehensive programs with longer duration may be needed in order to effectively change parenting behavior. At the same time, our results indicate that programs should not provide services for too long. For example, a recent RCT ([35, 38]; not included in the meta-analysis, because maltreatment was not an outcome measure) indicated that standard PCIT with 12 sessions was equally effective or even significantly more effective for at-risk and maltreating families compared to time-variable PCIT with on average 17 sessions. More is not necessarily better.

Although the effectiveness of intervention programs is sometimes presented as promising (e.g., [1, 3]), the current meta-analytic results prove the opposite and seem to support a rather gloomy picture of the evidence base of widely used intervention programs. Several factors may contribute to this contrast. First, the main outcome measures in many effectiveness studies aimed at *preventing* child maltreatment are risk factors for child maltreatment (e.g., Child Abuse Potential Inventory, parenting stress), instead of actual maltreatment measures. Although risk factors are important and the reduction of these factors following the intervention program should be considered a positive sign, they do not provide information about the actual prevention or reduction of child maltreatment which is the goal of the interventions. For example, in the current meta-analysis, nearly 20 % of full text papers were excluded because child maltreatment was not an outcome measure. Moreover, some interventions were effective in improving parenting or child health, but did not effectively prevent or reduce child maltreatment. For instance, Jouriles and colleagues [28] found positive effects of Project Support on several problematic parenting variables, such as mothers' perceived inability to manage childrearing responsibilities, psychological distress, and observed ineffective parenting, whereas the number of subsequent reports to CPS did not differ between the experimental and control condition. Second, in some RCTs, effects were found in specific subgroups, which were not fully randomized as such. For example, Olds and colleagues [55] conclude that their Nurse-Family Partnership has a significant effect on child maltreatment at direct follow-up for pregnant women with each of three risk factors. However, the original randomized sample included pregnant women of whom 85 % had *at least one* of the three risk factors and only 23 % had each of the three risk factors. There was no significant effect when the whole sample was included in the analyses. Such subgroup analyses indicate that the results are no longer based on a true RCT, and therefore may yield inflated effect sizes that have to be replicated in independent trials randomized with stratification across number of risk factors.

Sample size and year of publication were both significant and independent predictors of the effect size in a multivariate approach. Effect sizes were smaller with increasing sample size and larger effect sizes were found for later studies, which may indicate that intervention programs improve or become more fine-tuned over time. Meta-analytic findings from Piquart and Teubert [22] indicated the opposite effect, with later studies yielding smaller effect sizes. This result was different from what the authors expected, and they argued that the general knowledge about parenting has increased over time, which reduces the size of possible intervention effects. In addition, they suggest that publication bias towards significant findings was a problem in the earlier years. The fact that we did find a positive effect for year of publication may be caused by the fact that we included studies that were published up until 2013, whereas Piquart and Teubert [22] only included studies published up until 2009. After controlling for sample size and year of publication only focus of the program remained a significant predictor of program effectiveness.

### Limitations

The current meta-analysis is based on a rather small number of studies. Part of the moderator analyses should therefore be considered as exploratory. Whereas the full sample consisted of a set of 27 effect sizes, several moderator analyses were based on a relatively small number of effect sizes. For example, only six studies included more than one follow-up. More long term follow-ups are needed to obtain more robust findings about possible effects of programs to prevent and reduce child maltreatment on the long term.

In addition, some characteristics lacked variance to test for moderating effects. Given the small number of RCTs conducted outside the USA, the possibility to examine the moderating effect of continent of origin was limited. In the same vein, it was not possible to examine the effect of type of intervener, since all intervention programs that were included in the current meta-analysis were delivered by (para)professionals and none by other interveners such as laymen. Furthermore, programs targeting the general population were excluded from moderator analyses, since only one such study was included in the meta-analysis. Finally, many studies included in the meta-analysis only included an overall measure of maltreatment or only one type of maltreatment, mostly physical abuse. Therefore, it was impossible to examine program effectiveness on different types of maltreatment. In order to disentangle the effect of intervention programs more thoroughly, and thus to provide the most appropriate services to different families, RCTs should separately report on different types of maltreatment.

### Future directions

This meta-analysis implies several possibilities for improvement of program effectiveness studies. First, we clearly need more RCTs that examine the effect of intervention programs on the prevention or reduction of child maltreatment, also outside the USA and in low- and middle-income countries. Nearly half of all full text papers that were screened for eligibility had to be excluded because no RCT was done. Indeed, it has been argued that it is too difficult for practical and ethical reasons to conduct RCTs with maltreating or at-risk families [60], and this may be even more so for low- and middle-income countries. However, the fact that we did find six RCTs in maltreating families and 20 RCTs in at-risk families, including one study in Iran, indicates that it is possible to carry out rigorous effectiveness studies in various populations. Only RCTs can strengthen the evidence base for maltreatment interventions needed so badly in practice.

Moreover, effectiveness studies of intervention programs that aim to reduce or prevent child maltreatment should take child maltreatment as their primary outcome measure. As noted above, many studies mainly focus on parenting behaviors or risk factors for child maltreatment, while actual changes in child maltreatment seem somewhat neglected. When child maltreatment measures are used as main outcome variables, a multimethod approach is advisable (e.g., [6]). Previous meta-analytic evidence indicates that reports from professionals yield considerably lower prevalence rates than self-report measures [4]. Therefore, effect sizes of intervention programs will be more reliable when more than one method is used to examine how often child maltreatment occurs [61]. In addition, for intervention programs that do effectively reduce child maltreatment, future studies should examine whether they are also effective in reducing the number and duration of out-of-home placements.

Finally, future research should focus on the development and testing of prevention programs. Results of our meta-analysis indicate that so far intervention programs are only effective in *reducing* child maltreatment, and thus only protect children when the harm has been done. From all programs in the current meta-analysis that aim to *prevent* child maltreatment, only one program was universal [14], while 19 other programs targeted a high-risk population. However, prevention efforts should not only focus on populations with the highest risk for maltreatment. From all maltreating families, only a small proportion belongs to this group of high risk families [62]. In order to protect all children against maltreatment, the development and testing of both universal and targeted prevention programs should be prioritized.

## Conclusions

Findings from the current meta-analysis indicate that intervention programs have no overall significant effects on the reduction or prevention of child maltreatment, when publication bias against smaller studies with non-significant results is taken into account. Significant effects were however found for intervention programs reducing child maltreatment in maltreating families and for interventions that provide parent training. More RCTs are needed to strengthen the evidence base of program effectiveness, especially in the field of prevention.

## Additional file

Additional file 1:
**PRISMA checklist.** (DOC 62 kb)
